# New Trends in C–C Cross-Coupling Reactions: The Use of Unconventional Conditions

**DOI:** 10.3390/molecules25235506

**Published:** 2020-11-24

**Authors:** Marta A. Andrade, Luísa M. D. R. S. Martins

**Affiliations:** Centro de Química Estrutural and Departamento de Engenharia Química, Instituto Superior Técnico, Universidade de Lisboa, 1049-001 Lisboa, Portugal; marta.andrade@tecnico.ulisboa.pt

**Keywords:** cross-coupling reactions, microwave irradiation, ultrasounds, mechanochemistry, solvent-free, water, ionic liquids, deep eutectic solvents, sustainability

## Abstract

The ever-growing interest in the cross-coupling reaction and its applications has increased exponentially in the last decade, owing to its efficiency and effectiveness. Transition metal-mediated cross-couplings reactions, such as Suzuki–Miyaura, Sonogashira, Heck, and others, are powerful tools for carbon–carbon bond formations and have become truly fundamental routes in catalysis, among other fields. Various greener strategies have emerged in recent years, given the widespread popularity of these important reactions. The present review comprises literature from 2015 onward covering the implementation of unconventional methodologies in carbon–carbon (C–C) cross-coupling reactions that embodies a variety of strategies, from the use of alternative energy sources to solvent- free and green media protocols.

## 1. Introduction

Cross-coupling reactions have attracted and inspired researchers in the academia and industry for decades, given its significance as a synthetic tool in modern organic synthesis. The continuous interest in cross-coupling reactions, with more than 40 years of history, has been mostly driven by its valuable contributions and applications in the medicinal and pharmaceutical industries. Especially noteworthy are reactions furnishing carbon-carbon bonds, regarded as one of the most challenging tasks in organic synthesis. C–C Cross-coupling protocols developed by Suzuki [1], Heck [2], and Sonogashira [3], among others, changed the way in which organic synthesis is conceptualized, endowing a practical synthetic route for the direct formation of C–C bonds [4,5,6]. 

The continuous challenge of the design of reactions from a sustainable viewpoint (e.g., more efficient energy consumption, easy catalyst recovery, avoidance of high amounts of organic solvents) has led to the development of greener reaction protocols [7,8,9,10]. Tremendous efforts have been paid to develop alternative energy inputs that can dramatically reduce the reaction time, resulting in higher yields of the desired product. On the other hand, the use of eco-friendly media or solvent-free protocols is preferred over common organic solvents to obtain the maximum conversion in an eco-friendly manner [11,12,13]. Other issues such as efficient separation and subsequent recycling of the catalyst have been effectively solved, mostly by heterogeneous catalytic systems, many of them involving metallic nanoparticles [14,15], and, also, by the development of magnetic-supported catalysts [16,17,18].

The implementation of green methodologies in C–C cross-coupling reactions relies on a variety of strategies, such as the use of microwave irradiation, ultrasounds, and mechanochemistry as energy input under green and sustainable reaction media [11,19]. In this review, we wish to cover recent advances reported in the literature from 2015 to date that have demonstrated a special focus on unconventional methodologies for the generation of C–C bonds using cross-coupling reactions. Out of the scope of this review are photo-induced cross-coupling reactions. The synthesis of novel heterogeneous catalysts is not analyzed separately. 

## 2. Alternative Activation Methods for Cross-Coupling Reactions

The use of alternative means of activation can improve many established protocols, providing superior results when compared to reactions performed under conventional conditions. Microwave (MW) irradiation, ultrasounds, and mechanochemistry can be used to enhance conventional experimental techniques for cross-coupling reactions.

### 2.1. Microwave Irradiation

Microwave (MW)-assisted synthesis has developed into a popular branch of synthetic organic chemistry, being currently employed for a variety of chemical applications. MW irradiation produces efficient internal heating by direct coupling of MW energy with the molecules (solvents, reagents, and catalysts) that are present in a reaction mixture, in contrast to conventional heating methods [14,20,21]. Reaction vessels employed in MW reactors, typically made out of microwave transparent materials (borosilicate glass, quartz, or Teflon), allow the radiation to pass through the walls of the vessel, creating an inverted temperature gradient as compared to conventional thermal heating [22]. This unconventional MW energy results in the acceleration of chemical reactions due to the selective absorption of MW radiation by polar molecules [20,21,23]. The rate enhancements are essentially a result of a thermal effect, a change in temperature compared to heating by standard convection methods [24,25]. The use of MW irradiation fulfils the requirement of green chemistry, providing rapid heating, reduced reaction times, and, in many cases, increased yields and selectivity with significant energy savings [23,26]. MW-assisted reactions have been studied in detail by many researchers in numerous scientific areas, highlighting the advantages of this methodology [22]. High-yielding C–C cross-coupling reactions under the influence of MWs have been described in the literature for the past decade. There are extensive studies about Suzuki–Miyaura and, to a lesser extent, Mizoroki–Heck and Sonogashira-type C–C cross-coupling reactions incorporating MW irradiation.

**Scheme 1:**
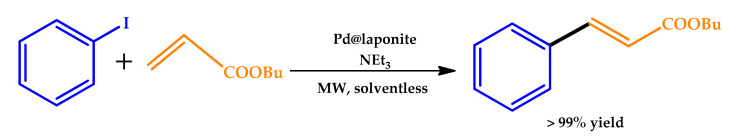
Heck reaction of iodobenzene with butyl acrylate using laponite-supported Pd nanoparticles (NPs) [27]. MW: microwave.

In 2015, Martínez et al. described the MW-assisted Mizoroki–Heck reaction, under solvent-free conditions, using a new family of solid catalysts, consisting in supported Pd nanoparticles (NP) on a synthetic clay (laponite) (Scheme 1) [27].

The influence of reaction time and MW power employed on the catalytic results was thoroughly studied by the authors. Complete conversions and excellent product yields were achieved in a few minutes, representing a remarkable increase of around 24 to 60 times in comparison to the use of conventional heating. It was observed that the time necessary to reach complete conversion was dependent on the power of the MW source. The catalyst was efficiently recovered and reused several times, achieving total conversion, outperforming most of the cases described in the literature. Nevertheless, the use of high MW power was found to be detrimental for the catalyst lifetime, leading to catalyst poisoning by coke deposition. In this sense, the authors highlighted that the best performances could be obtained using MW power up to 25 W.

**Figure 1:**
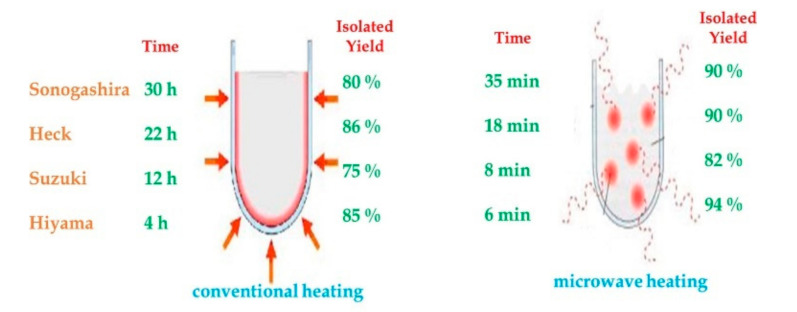
A comparison of time and yield for standard protocols of various coupling reactions. Reproduced with the permission of reference [28], Copyright 2016 Elsevier B.V.

Shah et al. provided an example of synergism between PdNP and MW heating in ligand-free C–C cross-coupling reactions—namely, Suzuki, Hiyama, Heck, and Sonogashira [28]. PdNPs < 10 nm were impregnated onto a commercially available microporous polystyrene resin and successfully used to synthesize asymmetric terphenyls by the sequential addition of aryl boronic acids. Highly pure coupled products were obtained in 6–35 min under MW heating, using benign low-boiling solvents that otherwise would involve 4–30 h of inert atmosphere under conventional heating (Figure 1). No side-products were observed, and, in most cases, the products were purified by simple crystallization. The catalyst was successfully recycled up to six times without significant leaching or a decrease in efficiency. A TEM analysis showed that the size of the NP remained unchanged after the reaction. 

Massaro and colleagues prepared a covalently linked thermo-responsive polymer on halloysite nanotubes (HNTs) by means of MW irradiation, used as a support and stabilizer for PdNP [29]. The prepared HNT-poly(*N*-isopropylacrylamide) (PNIPAAM) nanomaterial showed a good catalytic activity towards the Suzuki cross-coupling reaction of phenylboronic acid and several aryl halides under MW irradiation in aqueous media and low catalyst loading (0.016 mol%), reaching turnover numbers (TONs) and turnover frequencies (TOFs) up to 6250 and 37,500 h^−1^, respectively (Table 1). The catalyst was easily separated from the reaction mixture and reused for five cycles, with a minor drop in its catalytic activity and negligible Pd leaching.

**Scheme 2:**
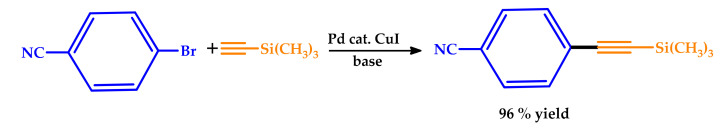
Microwave-assisted Sonogashira-type coupling of 4-bromobenzonitrile to 4-((trimethylsilyl)ethynyl)benzonitrile [30].

The beneficial use of MWs was also observed in a Sonogashira-type coupling reaction and reported by Lei et al. [30]. After the study of the effect of different catalysts, bases, solvents, temperatures, and reaction times on the coupling of 4-bromobenzonitrile to 4-((trimethylsilyl)ethynyl)benzonitrile, chosen as the model reaction (Scheme 2), the authors extended the scope of the MW-assisted reaction to a variety of substrates. Efficient conversion of the substrates into the desired 1-aryl-2-(trimethylsilyl)acetylene products with excellent yields was observed within a short reaction time under MWs. Compared to the 4-bromobenzonitrile substrate, the 4-iodobenzonitrile substrate also afforded quantitative yields. Moreover, several functional groups were well-tolerated under optimized conditions, with excellent yields. The use of MWs was found to significantly improve the efficiency of the method based on Cu and Pd-catalyzed Sonogashira-type coupling processes in a short reaction time, making it a useful approach for the synthesis of structurally diverse 1-aryl-2-(trimethylsilyl)acetylene.

**Scheme 3:**
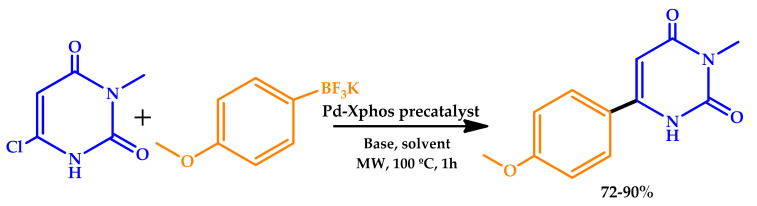
Suzuki–Miyaura cross-coupling reaction under MW irradiation [31].

Savitha et al. explored the MW-assisted Suzuki–Miyaura cross-coupling reaction of the chloro uracil analog with a wide array of (hetero) aryl potassium organotrifluoroborates, in water [31]. Aryl and heteroaryl trifluoroborates were found to be excellent reagents for Pd-catalyzed cross-coupling reactions with 6-chloro-3-methyluracil (Scheme 3). A set of electron-rich phosphines was employed, along with Pd(OAc)_2_, as the catalyst. Although none of the catalytic systems provided exceptional results, the catalytic activity was significantly enhanced when a Pd-XPhos precatalyst, a coordinatively unsaturated complex, was employed, with excellent conversions (72–90%). The screening of solvents showed that the reaction afforded exceptional conversion in plain water, which, in combination with MW irradiation, increased its green potential. 

In 2017, Baran and coworkers designed a heterogeneous Pd(II) catalyst containing a *O*-carboxymethyl chitosan Schiff base (CS-NNSB) as the support material. The catalytic activity of CS-NNSB-Pd(II) was examined in Suzuki cross-coupling reactions under mild conditions using a simple MW heating technique and solvent-free reaction media [32]. CSNNSB-Pd(II) presented a high catalytic performance and excellent selectivity for the coupling reaction of phenyl boronic acid with 4-bromoanisol, with very low catalyst loading (6 mmol%) in a very short reaction time (5 min), offering high TON and TOF values (up to 16,167 and 202,087), confirming the suitability of the proposed catalytic system. The substrate scope was extended under the optimum conditions with a range of aryl halides, containing withdrawing groups or electron-donating functional groups. While the coupling reactions of aryl bromides and iodides provided the desired biaryls products with excellent yields, relatively lower reaction yields were observed for the aryl chlorides owing to the poor reaction activity. In addition, the reusability and catalytic behavior of the catalyst was tested, and it could be reused up to seven runs. Verbitskiy and colleagues proposed a convenient method for the synthesis of a new fluorophore on the basis of 4,5,6-tri(het)-arylpyrimidine containing three electron-donating carbazole moieties by consisting of a series of MW-assisted Suzuki cross-coupling reactions [33]. The synthesis of the target structure of fluorophore was accomplished by using the sequence of MW-assisted Suzuki cross-coupling and bromination with N-bromosuccinimide, using as a starting material 5-bromo-4,6-di(thiophen-2-yl)pyrimidine. The reaction time never exceeded 25 min, and the yield of the target fluorophore 3,3’-(5,5’-(5-[5-(9-ethyl-9H-carbazol-3-yl)-3-hexylthiophen-2-yl]pyrimidine-4,6-diyl)bis(thiophene-5,2-diyl))bis(9-ethyl-9H-carbazole) reached 56%, with the 29% combined yield over three steps. The optical properties of the obtained compound were explored, envisioning possible applications in sensors for nitroaromatic compounds.

**Scheme 4:**
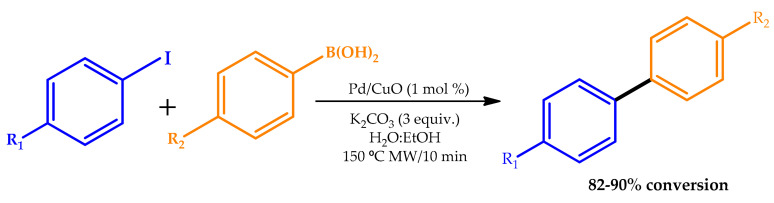
Suzuki cross-coupling reactions using a Pd/CuO catalyst [34].

MW irradiation was employed in another study for both the preparation of the catalyst and its application for the Suzuki cross-coupling reaction of bromobenzene and phenylboronic acid [34]. Elazab et al. developed a simple and efficient synthetic protocol to produce highly active PdNP catalysts supported on a copper oxide matrix using MW irradiation through a simple and fast approach under mild temperature and pressure (Scheme 4). The synthesized Pd/CuO bimetallic catalyst with an average size of 20–40 nm was investigated towards the Suzuki cross-coupling reactions in 50% aqueous ethanol solvent using MW irradiation.

The prepared Pd/CuO catalyst was found to be stable, showing excellent conversion within 15 min at 150 °C, highlighting the crucial role played by the CuO solid support in preventing NP agglomeration. Moreover, it could be simply recovered and recycled up to five times with a negligible loss in performance or catalytic activity under the batch reaction. The implemented MW irradiation method was recognized as simple, reliable, and rapid, allowing the synthesis of controlled-size NPs. The results obtained for the Suzuki cross-coupling reaction are in line with those observed by Massaro et al. [29] and discussed above in this section, even though the comparison is not straightforward, as the experimental conditions are distinct—namely, catalyst support and loading and the microwave power employed. 

Zakharchenko et al. described the efficient MW-assisted C–C cross Suzuki–Miyaura coupling reaction with a series of catalysts, including Pd(II) complexes. The synthesized materials, consisting of 3-(2-pyridyl)-5*R*-1,2,4-triazoles Pd(L^R^)_2_ (R = ethyl, *n*-propyl, *i*-propyl, and *t*-butyl) catalyzed the MW-assisted Suzuki–Miyaura cross-coupling reaction of bromoanisole with phenyl boronic acid in the presence of a base. Furthermore, these heterogeneous catalysts were recovered and reused without losing activity for quite a few consecutive cycles [35]. The low power of the MW irradiation (10 W) used provided a much more efficient synthetic method than conventional heating, allowing for the attainment of significantly better yields in much shorter times. 

### 2.2. Sonochemistry

Ultrasound-promoted organic synthesis is a powerful and important green methodology. Sonochemistry is the use of ultrasound irradiation in chemical processes, leading to the generation of intense mechanical, thermal, and chemical effects in a liquid medium as the consequence of the cavitation phenomenon throughout the generation, growth, and sudden collapse of gaseous microbubbles [14,36]. This feature creates extremely high local pressures (up to 1000 bar) and high temperatures (up to 5000 K) that can trigger high-energy radical mechanisms in mild conditions [24]. The physical properties of the irradiated mixture are crucial for the effectiveness of cavitation, as well as for the proper transfer of acoustic energy to the reactants.

Ultrasounds can therefore be used as an important tool to perform a number of chemical reactions, enhancing rates and yields at shorter reaction times, with simpler and easier workup conditions compared to conventional procedures [26,36]. Ultrasound-promoted procedures that combine sonochemistry with green, nonconventional solvents or even solvent-free have been increasingly reported in the literature, including, most frequently, the use of water, ionic liquids, poly(ethylene glycol) (PEG), and others. Despite the large and diverse number of reports on this topic, very few data are available on comparative results obtained for direct and indirect sonochemistry procedures. 

**Scheme 5:**
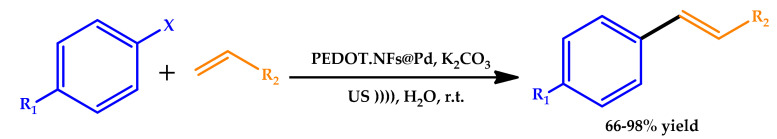
US-assisted Mizoroki–Heck cross-coupling reactions [37]. NF: nanofibers.

In 2017, Rezaei developed a highly efficient procedure involving PEDOT nanofibers/Pd(0) composites (PEDOT-NFs@Pd) as recyclable catalysts for the C–C cross-coupling reactions of aryl halides with olefinic compounds under ultrasonic irradiation in water (Scheme 5) [37]. The catalytic performance of the catalyst toward Heck cross-coupling reactions under ultrasound irradiation was examined, being clear that the coupling reaction could be finished in a shorter time with better yields. The author further explored the catalytic applicability of PEDOT-NFs@Pd towards Heck coupling reactions of styrene and n-butyl acrylate with different aryl halides under optimized conditions, showing that various aryl bromide or iodide with different substituent groups could be efficiently converted to the corresponding products in high yields. Moreover, the catalyst was recovered by simple filtration and reused quite a few times without a significant loss of activity.

Panahi and colleagues reported the synthesis of a Pd nanomaterial based on a Cu-metal-organic framework (MOF), Pd@Cu-MOF, as an effective catalyst for a Suzuki–Myaura C-C cross-coupling under ultrasonic irradiation [38]. Operational parameters, such as temperature, time, solvent, and base, were the first objects of optimization in a model reaction of phenylboronic acid and p-bromobenzene. Subsequently, different derivatives of biaryl compounds were synthesized by the US-assisted reaction of different aryl halide derivatives and phenylboronic acid derivatives in shorter reaction times and considerably higher yields (75–99%) in the presence of a base. The catalyst was recoverable, being reused for at least four consecutive reaction cycles. 

**Figure 2:**
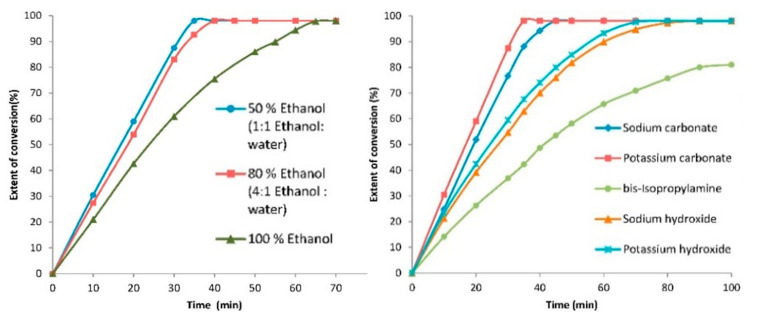
(**Left**): Effect of solvent combination on the US-assisted Suzuki cross-coupling reaction. (**right**): Effect of different bases on the US-assisted Suzuki cross-coupling reaction. Reaction Conditions: 4-bromoanisole (1 mmol) and phenylboronic acid (1.5 mmol) using 1.5 mol% of catalyst Pd/C, base (2.0 mmol), 30 °C, ultrasonic bath, 22 kHz, and the irradiation power of 40 W. Adapted with the permission of reference [39], Copyright 2017 Elsevier B.V.

In 2018, there was a US-assisted and Pd-catalyzed reaction of 4-bromoanisole and phenylboronic acid in a 1:1 mixture of ethanol and water [39]. The authors explored the effects of several operating parameters, such as US power, temperature, catalyst loading, and molar ratio on the catalytic performance (Figure 2). 

Overall optimum conditions established were the catalyst loading of 1.5 mol%, molar ratio of (phenylboronic acid:4-bromoanisole) 1.5, US power of 40 W, and duty cycle of 90% at a frequency of 22 kHz for a maximum conversion of 98%, achieved in 35 min. This study also demonstrated a very good catalytic performance using a volume ten times higher as compared to the normally used volumes in the case of a simple ultrasonic horn. It was also observed that the reaction rate increased with an increase in temperature over the range of 30–60 °C, decreasing beyond this temperature. 

The development of a simple US-assisted protocol for the Suzuki–Miyaura reaction yielding biphenyl compounds was reported by Baran et al. [40]. The catalytic performance of PdNPs@CMC/AG, consisting of Pd(0)NP on a natural composite composed of carboxymethyl cellulose/agar polysaccharides (CMC/AG), was evaluated in the synthesis of various biphenyl compounds by using ultrasounds. 

The prepared nanocomposite exhibited an excellent catalytic performance for cross-coupling reactions that contained aryl iodides, achieving high reaction yields. In addition, PdNPs@CMC/AG was successfully reused up to six reaction cycles without a loss of its catalytic activity, indicating a high reproducibility of PdNPs@CMC/AG. Compared to conventional methods, the US-assisted synthesis technique reported in this study exhibited some advantages, such as a shorter reaction time, greener reaction conditions, higher yields, and easier workup.

In 2018, Naeimi et al. reported a novel and efficient catalytic system consisting of Cu(I)-immobilized on functionalized graphene oxide for the Sonogashira coupling reaction under ultrasonic irradiation [41]. The prepared nanocatalyst GO@SiO_2_-HMTA-Cu(I) (GO: graphene oxide; HMTA: hexamethylenetetramine) afforded excellent yields of diarylethene compounds and short reaction times under mild reaction conditions (Table 2). The catalytic activity of GO@SiO_2_-HMTA-Cu(I) was found to be dependent on the type of leaving group: all aryl halides (chloride, bromide, and iodide). The products can be easily separated from the reaction mixture by filtration. Furthermore, the catalyst was simply recycled and reused eight times without a significant loss of activity. 

In 2019, the same authors reported the synthesis of a magnetic thiamine Pd complex nanocomposite, a novel, highly active, and reusable catalyst for the Mizoroki–Heck coupling reaction of several types of iodo, bromo, and even aryl chlorides under US irradiation [42]. The prepared Pd catalyst demonstrated an excellent performance for the efficient, mild, fast, clean, and safe sonochemical synthesis of trans-stilbene derivatives at room temperature, producing the corresponding trans-coupled products in excellent yields and moderate reaction times. The authors first examined the efficiency of the solvent in the Mizoroki–Heck reaction, DMF being the most efficient for this transformation. The gathered data demonstrated that, in the presence of ultrasonic irradiation and 0.02 g of the Pd(0) catalyst, the reaction time was shortened to 8 min, with a sharp increase of the reaction yield to 99% (Table 3). The catalyst was separated by the use of an external magnet and reused several times without a marked loss of activity. 

### 2.3. Mechanochemistry

A mechanochemical reaction is defined as a chemical reaction that is induced by the direct absorption of mechanical energy [43]. Mechanochemical processes are completely different from thermal processes, being usually performed using grinding in ball mills. The high speed achieved under ball milling conditions results in a homogeneous mixture of reactants, which successively facilitates chemical reactions. Particle refinement leads to an increase of surface area, surface energy, and number of defects. Mechanochemistry can be used for the mechanical activation of solids, due to the increased surface energy, alterations in structure, chemical composition, or chemical reactivity occurring throughout the milling process. The interest in mechanochemical synthesis is growing at a fast pace, as it provides convenient, feasible, and greener methods for the synthesis of valuable chemical compounds.

This method has been successively applied to several organic chemical reactions, involving many methodologies under solvent-free conditions and, also, finding enhanced selectivities and reactivities compared to reactions performed in a solution. Examples of mechanochemical-assisted cross-coupling reactions are now becoming notorious in the literature.

**Figure 3:**
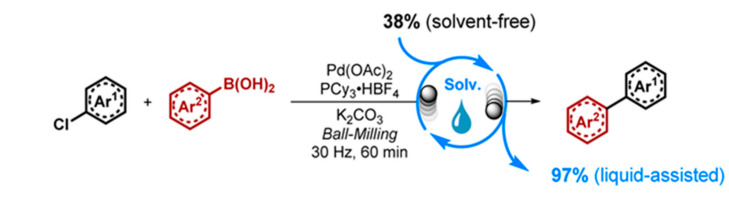
Mechanical Suzuki−Miyaura reaction of aryl chlorides using liquid-assisted grinding. Reproduced with the permission of reference. [44], Copyright 2016 American Chemical Society.

In 2016, Jiang et al. studied the effects of the liquid-assisted grinding in the mechanochemical Suzuki−Miyaura reaction of aryl chlorides [44]. The catalytic systems, using Davephos or PCy_3_, were tested, showing strong influences from different liquids, with an unexpected improvement of the yield over 55% when using alcohols as additives, which is most likely explained by in-situ formed alkoxides and their participation in the oxidative addition. Further expansion of the substrate scope using the Pd(OAc)_2_/PCy_3_/MeOH system afforded the desired products in good-to-high yields, proving that it can be applied to both activated and unactivated aryl chlorides with good-to-high yields (Figure 3). The result implicates that the liquid-assisted grinding effect does not only provide a sub-stoichiometric solvent environment but may also participate in the formation of mechanically induced transition species. Thus, this technique may have the potential to induce higher catalytic activity for existing systems under high-speed ball milling conditions.

The result implicates that the liquid-assisted grinding effect does not only provide a sub-stoichiometric solvent environment but may also participate in the formation of mechanically induced transition species. Thus, this technique may have the potential to induce a higher catalytic activity for existing systems under high-speed ball milling conditions.

In 2017, Shi et al. described the synthesis of a cheap and reusable Pd catalyst supported on MgAl-layered double hydroxides (Pd/MgAl-LDHs) to be used in Heck reactions under high-speed ball milling conditions at room temperature [45]. The authors explored the effects of the ball milling size, ball milling filling degree, reaction time, rotation speed, and grinding auxiliary category in the yields of mechanochemical Heck reactions. The obtained results point out the suitability of this catalyst for high-speed ball milling (HSBM) systems, affording a wide assortment of Heck coupling products in satisfactory yields. Moreover, the catalyst could be easily recovered and reused for at least five times without a significant loss of catalytic activity.

**Scheme 6:**
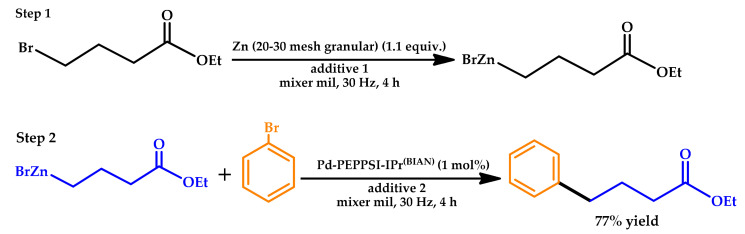
Optimization of the one-jar, two-step reaction for mechanochemical Negishi cross-coupling reactions [46]. PEPPSI: pyridine-enhanced pre-catalyst preparation stabilization and initiation.

Cao et al. reported in 2018 a novel mechanochemical method for the synthesis and subsequent reaction of organozinc species in a Negishi cross-coupling reaction [46]. Organozincs were generated from an alkyl halide with the addition to the reaction mixture of a coupling partner along with a Pd catalyst and tetrabutylammonium bromide (TBAB) to perform the Negishi reaction in a one-pot, two-step process (Scheme 6). 

The approach showed a broad substrate scope, offering broad opportunities for the in-situ generation of organometallic compounds from base metals and their simultaneous engagement in mechanochemical synthetic reactions without the need to use inert atmosphere techniques or dry solvents.

Seo et al. reported a very complete study on the development of a broadly applicable mechanochemical protocol for the solid-state Pd-catalyzed organoboron Suzuki–Miyaura cross-coupling reaction [47]. The authors studied the influence of olefin additives that might act as molecular dispersants and the effect of the phosphine ligand on the solid-state organoboron cross-coupling reaction. The influence of the mechanochemical reaction parameters on the solid-state reaction of 4-bromo-1,1’-biphenyl with 4-dimethylaminophenylboronic acid was also investigated (Table 4). Aiming to explore the scope of the present solid-state coupling reaction, a variety of solid aryl bromides was used, affording good-to-excellent yields (%). 

**Figure 4:**
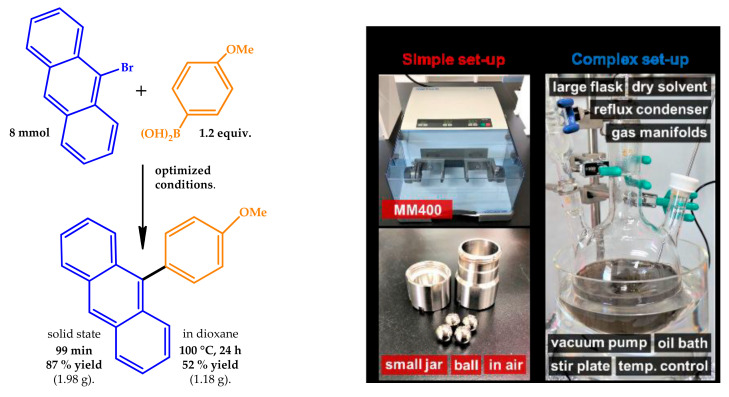
(**Left)**: Reaction scheme for the solid-state cross-coupling of 9-bromoanthracene with *p*-methoxyboronic acid. (**Right**)**:** Solid-state coupling reaction on the gram scale. Reproduced with the permission of reference [47], Copyright 2019 Royal Society of Chemistry.

Additionally, the use of solid aryl chlorides, boronic acids, aryl dibromides and dichlorides, and polyaromatic hydrocarbon substrates were also studied for this reaction. Furthermore, the authors explored the synthetic utility of the proposed protocol, conducting the solid-state cross-coupling reaction on the gram scale under mechanochemical conditions (Figure 4) of 9-bromoanthracene with 4-dimethylaminophenylboronic acid at the 8.0-mmol scale in a stainless-steel ball milling jar using four stainless-steel balls, affording 87% yield in 3 h.

**Figure 5:**
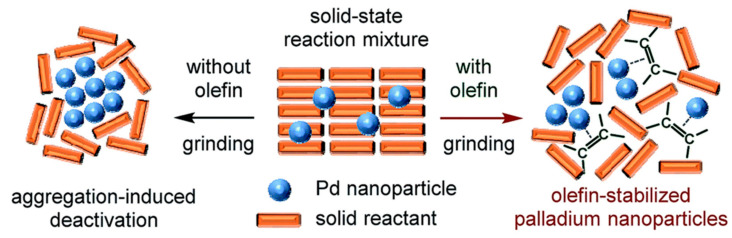
Possible roles for the olefin additives in solid-state cross-coupling reactions. Reproduced with the permission of reference [47], Copyright 2019 Royal Society of Chemistry.

For comparison, a solution-based Suzuki–Miyaura cross-coupling reaction was also conducted in a large amount of solvent (dioxane) at a high temperature (100 °C) and extended reaction time (24 h), obtaining, in this case, a moderate yield (52%). The mechanistic data obtained suggested that olefin additives could act as dispersants for the Pd-based catalyst suppressing the higher aggregation of the NPs, which could lead to the catalyst deactivation, acting also as stabilizer for the active Pd(0) species upon coordination (Figure 5) [47]. 

**Figure 6:**
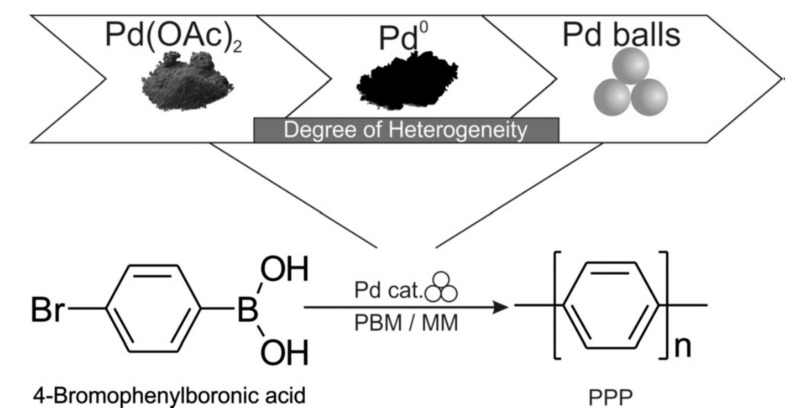
Mechanochemical Suzuki polymerization reaction of 4-bromophenylboronic acid to poly(para-phenylene) (PPP). The catalyst was simplified from a Pd salt to Pd black and to simply Pd milling balls. PMB: planetary ball mill; MM: mixer ball mill. Reproduced with the permission of reference [48], Copyright 2019 Wiley-VCH Verlag GmbH & Co. KGaA, Weinheim, Germany.

Vogt et al. introduced a facile and highly sustainable synthesis concept for a Pd-catalyzed mechanochemical reaction, turning catalyst powders, ligands, and solvents obsolete (Figure 6). The authors performed the Pd-catalyzed C-C Suzuki polymerization of 4-bromo or 4-iodophenylboronic acid, providing poly(para-phenylene) [48], and were surprised to observe one of the highest degrees of polymerization (199) reported in the literature. The solvent-free environment of a ball mill enabled the direct use of Pd milling equipment or Pd black catalyst, instead of conventional Pd(II) salts or Pd complexes. With 4-iodophenylboronic acid as the monomer, a good yield and high degree of polymerization were achieved in planetary ball milling using Si_3_N_4_ milling material, while full conversion to long-chain polymers was obtained in a mixer ball mill using a softer poly(methyl methacrylate) PMMA vessel. In addition, the degree of polymerization achievable by this method surpassed those obtained by solution or electrochemical processes. The obtained results indicate a most likely heterogeneous reaction that was not improved by using established ligands from solution-based homogeneous procedures.

Pentsak et al. investigated the solid-state reaction of aryl halides with arylboronic acids in the absence of a solvent and without any liquid additives [49]. A few important conditions for performing the Suzuki–Miyaura reaction were analyzed in detail, pointing out the prominent role of water, formed as a by-product in the side reaction of arylboronic acid trimerization. Minimal amounts of water allow the initiation of the reaction with potassium tetraphenylborate and potassium trifluoroborate. Electron microscopy studies revealed surprising changes that occurred within the reaction mixture, such as the formation of spherical nano-sized particles containing the reaction product. Catalyst recycling was easily performed, involving the product isolation by sublimation and, thus, providing the possibility to completely avoid the use of solvents at all stages (Table 5). Under optimized conditions, the quantitative yields could be achieved by conventional heating of the solid-phase reaction mixture, without the need for additional mixing. The PdNPs/multi-walled carbon nanotubes (MWCNT) catalyst can be reused multiple times without a loss of efficiency. The absence of a liquid phase is an important factor to avoid metal leaching.

Soliman and co-workers explored the preparation of Pd(II) and Pt(II) composites with activated carbon (AC), graphene oxide, and multiwalled carbon nanotubes by ball milling and their use as catalysts for the mechanical-assisted Suzuki–Miyaura reaction [50]. The Pd-AC composites exhibited high catalytic activity towards the mechanochemical cross-coupling of bromobenzene and phenylboronic acid, with yields up to 75%, achieving a TON and TOF of 222 and 444 h^−1^, respectively, using the Pd(4.7 wt%)-AC catalyst (Table 6). The effect of small amounts of olefins on the reaction yield was investigated for the most promising catalytic system (using cyclooctene as the additive), obtaining relevant yields of ca. 80%. The authors highlighted the importance of ball milling as a promising synthetic tool under eco-friendly conditions, in comparison to other approaches for the energy input, such as MW and conventional heating. 

## 3. Unconventional Media for Cross-Coupling Reactions

The use of organic solvents as the traditional medium for organic synthesis continues to be extensively applied. From a sustainable point of view, unconventional and more environmentally benign reaction media should be explored [9,51]. Widespread effort has been made to develop efficient catalytic systems under alternative media for cross-coupling reactions. 

### 3.1. Solvent-Free

According to the principles of green chemistry, solvent-free protocols for organic reactions are of great interest, reducing or eliminating the utilization of hazardous and expensive organic solvents. Solvent-free methodologies have been commonly used under MW irradiation or mechanochemical conditions often using supported reagents, achieving maximum conversion and minimizing wastes.

Dumonteil et al. described a Pd-catalyzed Mizoroki–Heck cross-coupling performed in solvent and ligand-free conditions for the synthesis of abscisic acid (ABA), a well-known phytohormone of great biological interest [52]. Upon optimization of the reaction parameters, various dienes and trienes were obtained in moderate-to-good yields without isomerization. The optimized solvent-free Mizoroki–Heck reaction was, furthermore, successfully applied to the synthesis of ABA, offering a short new pathway using environmentally friendlier solvents and reagents as an alternative to the synthesis already described in the literature.

Baran et al. devised a highly efficient heterogeneous Pd catalyst prepared using guar gum (GG), a natural biopolymer [53]. The catalytic studies showed that GG-Pd is a very active catalyst for Suzuki cross-coupling reactions under solvent-free media without any additives. Aryl halides bearing electron-attracting or donating groups were converted to the desired coupled product in excellent yields within 5 min under green reaction conditions, showing significant tolerance against different functional groups. Furthermore, the catalytic results indicated the yield order of biaryl yields as follows: Ar-I > Ar-Br > Ar-Cl (Table 7). 

Additional stability tests showed that the catalyst could be reused at least ten times, with a minor decrease of its catalytic performance and negligible Pd leaching. The authors foresaw the general applicability of the designed GG-Pd catalyst and in industrial applications due to its economic and catalytic advantages.

In 2019, Gribanov and colleagues reported the environmentally friendly and efficient synthesis of fully substituted 1,2,3-triazoles comprising a solvent-free Pd-catalyzed Suzuki cross-coupling reaction [54]. The efficiencies of the Stille and Suzuki reactions of halo-1,2,3-triazoles with pinacol arylboronates were compared, revealing the preference of the Suzuki method. The elaborated protocol proceeded without the use of solvents in aerobic conditions, low catalyst loadings, and KOH as a base. The authors demonstrated the wide scope of this methodology, which can be extended to heteroaromatic halides—particularly, challenging 4- and 5-halo-1,2,3-triazoles. 

**Figure 7:**
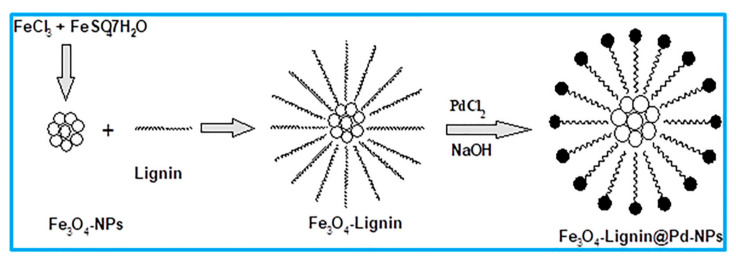
Synthesis of Fe_3_O_4_, Fe_3_O_4_-lignin, and Fe_3_O_4_-lignin@Pd-NPs. Reproduced with the permission of reference [18], Copyright 2020 John Wiley & Sons, Ltd.

Bharamanagowda and colleagues synthesized a new hybrid core shell catalyst, Fe_3_O_4_-lignin@PdNPs, through the support of PdNPs in a Fe_3_O_4_-lignin nanocomposite, obtained by sonication (Figure 7) [18]. The performance of this novel catalyst was evaluated towards the Mizoroki–Heck C-C cross-coupling reaction. The reaction between iodobenzene and n-butyl acrylate afforded a yield as high as 99% under solvent-free conditions. 

The scope of the catalyst was also explored for the same reaction of various aryl/heterocyclic halides and *n*-butyl acrylate/styrene under optimized conditions, attaining the respective products in high yields (73–99%). The catalyst was magnetically recovered and reused for five cycles of the Mizoroki–Heck reaction of iodobenzene and *n*-butyl acrylate, achieving yields of the desired products of 90–95% without a significant activity loss and without any contamination. No visible change was observed in the shapes and sizes of the PdNPs after recycling, as confirmed by TEM images.

### 3.2. Water as Solvent

Water is an abundant, safe, and green medium and has been applied to an array of organic reactions in the last two decades [10,55]. Water plays a dual role as a medium and as a cocatalyst in quite a few synthetic routes [9], playing an important role in the rate acceleration of reactions through the establishment of extensive hydrogen bonds with the functional groups of reactants, thus activating them in the process. In recent years, water has been used as a greener sustainable reaction medium and applied to cross-coupling reactions in different supported catalytic systems for a wide variety of nanoparticles as catalysts. The good yields might be due to a combination of the hydrophobic effect of water and the nature of the support and the stabilization of leached metallic reactive species in the water.

**Scheme 7:**
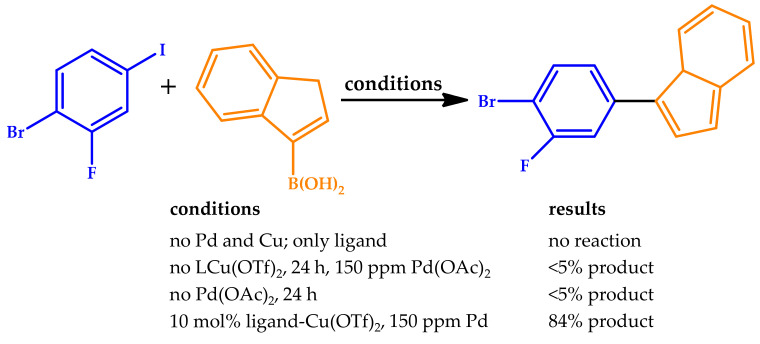
Control experiments confirming a synergistic effect in the Suzuki–Miyaura reaction of aryl iodides [56].

In 2016, Handa and co-workers proposed a new Cu/ppm Pd technology for selective Suzuki–Myaura reactions of aryl iodides carried out under the mildest conditions based on the remarkable synergistic effects between copper and ppm levels of Pd as the cocatalyst. Several biaryl couplings were run to investigate the scope of this process using Na_2_CO_3_ as the base, at 45 °C, the mildest conditions yet described for such Cu-catalyzed couplings, achieving, in general, moderate-to-good yields. Control experiments were performed to confirm the synergistic effects by Cu/ppm Pd (Scheme 7). [56]. The entire aqueous system, containing nanomicelles that function as the reaction medium, the base, copper, and Pd can be recycled. The yields for biaryl were all comparable throughout five cycles. The associated E_Factor_, an environmental factor that accounts for the ratio of the mass of waste per mass of product for these couplings was of about 5.8, ca. ten times lower than those reported and typically observed in the industry. 

Guarnizo and co-workers studied the formation of Pd/magnetite NPs through the anchorage of partially water-soluble 4-(diphenylphosphino)benzoic acid (dpa) on its surface [57]. The immobilized dpa enables the easy capture of Pd ions deposited on the surface of the magnetite nanoparticles after reduction with NaBH_4_. The catalytic efficiency for the Suzuki C–C coupling reaction was studied for Fe_3_O_4_dpa@Pdx, with different Pd loadings. The highest TOF was obtained for Fe_3_O_4_dpa@Pd0.5, affording the highest value reported to date for the reaction of bromobenzene with phenylboronic acid in a mixture of ethanol/water. Remarkably, the same reaction carried out in water also returned excellent yields. The small size of the PdNPs supported on magnetite in Fe_3_O_4_-dba@Pd0.3 and Fe_3_O_4_dba@Pd0.5, along with the presence of Pd single-atom catalysts, explains the excellent results for the Suzuki C–C coupling reaction both in neat water and in a mixture of ethanol/water.

In 2016, Ghazali-Esfahani et al. prepared a highly robust and active heterogeneous catalyst for Suzuki cross-coupling reactions that operated in water in the presence of an inexpensive base. For this matter, PdNPs immobilized on a cross-linked imidazolium-containing polymer were evaluated as a catalyst for Suzuki carbon-carbon cross-coupling reactions in aqueous media [58]. The prepared PdNP-polymeric ionic liquid (PIL) nanocatalysts showed good catalytic activities for aryl iodides and aryl bromides and moderate activity with aryl chloride substrates at low catalyst loadings (10 mg, 1.7 mol%). The coupling of sterically hindered substrates was also accomplished in reasonable yields (Table 8). Aryl iodides and aryl bromides were efficiently converted to biaryl products in the presence of phenylboronic acid in a high yield using short reaction times. The PdNP-PIL catalyst in water tends to be superior to various catalysts in ionic liquids media. Moreover, the PdNP-PIL catalyst is also able to couple aryl chloride substrates in a moderate yield (16–23%). The ability to reuse the PdNP-PIL catalyst was explored using two different aryl iodide substrates. For both substrates, good recyclability was observed over five catalytic runs with > 90% of the original activity retained with negligible leaching, confirming the high stability of the catalyst, which is rather unusual. The catalyst is simple to prepare, stable for prolonged periods, and easy to use and recycle.

Ahadi et al. reported the synthesis of novel Pd-supported periodic mesoporous organosilica based on the bipyridinium IL catalyst (Pd@Bipy–PMO) (periodic mesoporous organosilica) for the Suzuki–Miyaura coupling reaction in water [59]. The reaction of 4–bromoacetophenone with phenylboronic acid was chosen to perform the optimization of the overall process, studying the effects of the solvent, temperature, catalyst amount, and base. Furthermore, the stabilized Pd species inside the mesochannels provided high-to-excellent catalytic efficiency for the Suzuki–Miyaura coupling of various iodo– and bromo–aryl derivatives and different boronic acids, presenting excellent yields in a short reaction time. The activity of the heterogeneous catalyst was retained for six consecutive recycling runs.

The role of the base in the ligand-free Pd-catalyzed Suzuki–Miyaura reaction in water under mild conditions was explored by Wang et al. [60] through the establishment of a simple and efficient system. The tested bases were shown to stabilize active Pd species, preventing aggregation and deactivation of the species. For this matter, long-chain quaternary ammonium hydroxides were found to be the most suitable candidates. The effect of Pd loading on the reactivity of the reaction was studied by the authors (Table 9). The limited solubility of biaryl compounds in water allowed the development of an efficient method of filtration for product purification from the aqueous catalytic system. The ligand-free Pd-catalyzed Suzuki–Miyaura reaction showed improved durability in water with ppm levels of Pd loading. The entire catalytic system could be recycled after product separation. This water-compatible and air-stable effective catalytic protocol represented an attractive green synthetic improvement in Suzuki–Miyaura couplings.

**Figure 8:**
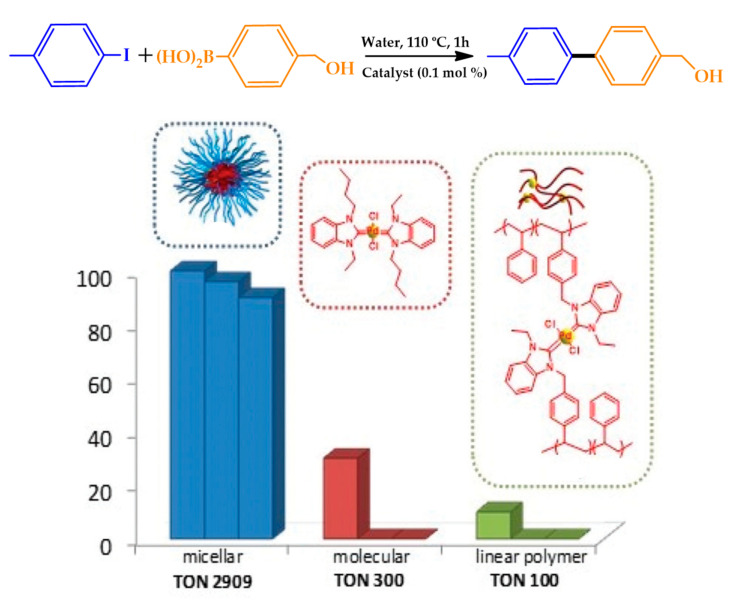
(**Top**)**:** Reaction scheme for Suzuki coupling of 4-(hydroxymethyl)phenylboronic acid and iodotoluene. (**Bottom**)**:** The three bars refer to the 1st, the 2nd, and the 3rd catalytic cycles, respectively. Reproduced with the permission of reference [61], Copyright 2018 The Royal Society of Chemistry. TON: turnover numbers.

Lambert et al. reported the highly efficient micellar catalysis in pure water provided by robust polymeric micelles consisting of Pd(II)–NHC (NHC: N-heterocyclic carbene) units [61]. The authors highlighted the several benefits conferred by the proposed approach to both the Suzuki–Miyaura and Heck cross-coupling reactions, such as a very broad substrate scope, outstanding catalytic activity in water, low catalyst loadings (0.1 mol%), easy recycling, and absence of metal leaching. For comparison purposes, a molecular compound and a copolymer both exhibiting an analogous structure were prepared separately and also tested (Figure 8). The three catalytic systems were compared for the Suzuki–Miyaura reaction of 4-(hydroxymethyl)phenylboronic acid and iodotoluene. Remarkably, the micellar catalyst led to full conversion, while the molecular and linear species provided 40% and 10% conversions, respectively. The substantial increase of the catalytic activity of the micellar catalyst in water was ascribed to the increase in the local concentration around the Pd(II)–NHC sites favored by the hydrophobic sequestrating effect.

All the features for the polymer are significantly distinct from the molecular and/or nonmicellar catalytic versions, affording promising nanostructured catalytic supports from a green chemistry point of view.

A sustainable and robust protocol for the copper-free Sonogashira cross-coupling under micellar aqueous reaction conditions using the commercially available catalyst CataCXium A Pd G3 was proposed by Jakobi et al. [62]. Several alkyne substrates were efficiently cross-coupled with a wide range of aryl halides, affording improved yields and low catalyst loadings. The Pd-catalyzed Sonogashira reaction (0.30 mol% catalyst loading) provided yields up to 98%, with the surfactant TPGS-750-M in H_2_O and THF as the cosolvent. Overall, the optimization of the reaction parameters rendered a simple and scalable operationally process able to provide a higher selectivity for heterocyclic compounds, alkynylated arenes, and monofunctionalized products achieved by the micellar aqueous reaction conditions.

### 3.3. Poly(Ethylene Glycol)

Poly(ethylene glycol) (PEG) oligomers are efficient, eco-friendly, and low-volatility liquids with a broad spectrum of chemical, industrial, and medical applications [63]. PEGs are readily miscible with water and polar organic solvents but immiscible with nonpolar organic solvents, aliphatic hydrocarbons, and supercritical CO_2_. The use of relatively inexpensive PEGs in recent years as a greener media in several organic reactions has gained considerable attention [19,64]. PEGs are suitable media for oxidation/reduction transformations, as they are nearly harmless, with a very low vapor pressure, high catalytic capacity, high thermal stability, and stability in both acidic and basic media. Furthermore, PEGs have been used as efficient additives in aqueous-phase cross-coupling transformations to improve the interaction between water-soluble catalysts and organic reactants.

**Scheme 8:**
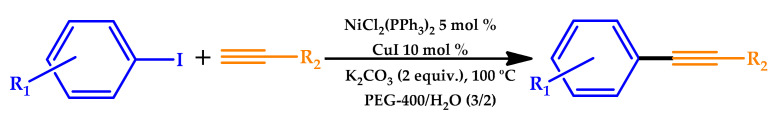
Nickel-catalyzed Sonogashira coupling in poly(ethylene glycol) (PEG-400)/H_2_O [65].

In 2015, PEG-400 was used as a solvent catalytic system for the Ni-catalyzed Sonogashira reaction by Wei et al. [65]. The coupling reaction of aryl iodides with terminal alkynes was carried out in a mixture of poly(ethylene glycol) (PEG-400) and water at 100°C with K_2_CO_3_ as the base in the presence of NiCl_2_(PPh_3_)_2_ and CuI, affording a range of arylacetylenes in good-to-excellent yields (Scheme 8). The recyclability of the NiCl_2_(PPh_3_)_2_/CuI/PEG-400/H_2_O system was assessed, allowing the recovery and recycling of the catalyst up to six times without a noticeable loss of catalytic activity. Apart from catalyst reuse, this system also avoided the use of easily volatile organic solvents.

PEG-400 was used a green solvent in the first Mn-catalyzed Sonogashira coupling reaction of aryl iodides under mild reaction conditions [66]. First, the reaction was performed with iodobenzene and phenyl acetylene as the model substrates in PEG-400 at 70 °C, using Mn(OAc)_2_2H_2_O as the catalyst and Et_3_N as the base. Different bases and other commonly applied Mn catalysts were also tested, as well as the effects of organic solvents, with no better results obtained compared with PEG-400. A variety of diarylacetylenes was the obtained in moderate-to-good yields under standard conditions using the proposed catalytic system. 

**Scheme 9:**
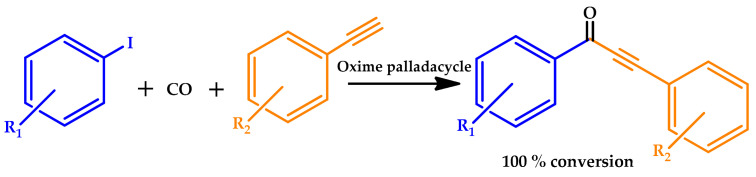
Oxime palladacycle-catalyzed carbonylative Sonogashira cross-coupling [67].

**Figure 9:**
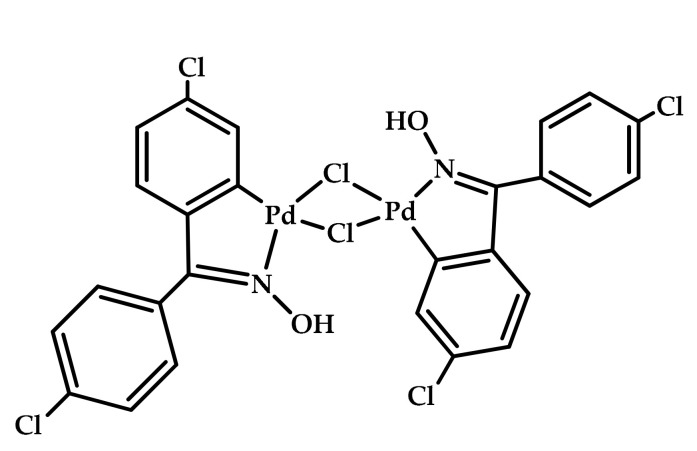
Oxime palladacycle used in the carbonylative Sonogashira cross-coupling of aryl iodides [67].

The use of PEG was also the choice of Gautam et al. as an environmentally benign solvent for the first palladacycle-catalyzed carbonylative Sonogashira cross-coupling of aryl iodides (Scheme 9) [67]. The oxime palladacycle (Figure 9) provided a phosphine-free approach for the synthesis of ynones at low Pd loadings, therefore resulting in high catalytic TONs and TOFs extremely higher than the best Pd catalyst reported in the literature for the model reaction of carbonylative cross-coupling between 4-iodoanisole and phenylacetylene. The oxime palladacycle used in this protocol behaved as an efficient and robust catalyst, thereby providing a phosphine-free protocol for the synthesis of ynones. The palladacycle catalyst showed a good recyclability, being reused up to four times with a slight decrease in activity.

**Figure 10:**
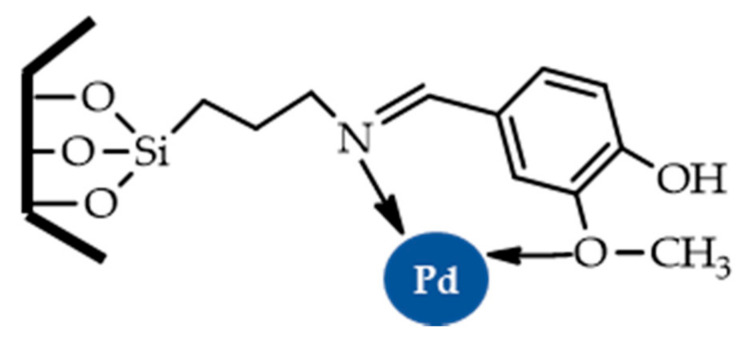
Structure of the Pd-vanillin-MCM-41 catalyst [68].

Khanmoradi et al. described the use of a Pd-vanillin Schiff base complex immobilized on mesoporous MCM-41 (Figure 10) as an efficient catalyst for the Suzuki–Miyaura, Stille, and Mizoroki–Heck reactions carried out in green solvents (H_2_O and PEG-400) [68]. 

The results indicated the Pd-vanillin-MCM-41 catalyst was a highly efficient catalyst for various aryl halides under mild conditions (Table 10). Phenyl iodides and phenyl bromides demonstrated a good reactivity, providing the corresponding products in good-to-very good yields for all reactions. The catalyst was reused for five consecutive cycles without a significant loss of its catalytic activity or metal leaching. 

Zeynizadeh and co-workers recently reported the use of PEG-400 as a green solvent for a Pd-free catalytic system in the Suzuki–Miyaura cross-coupling reaction [69]. A robust magnetic and reusable catalyst, Fe_3_O_4_@APTMS@Cp_2_ZrCl_x(x = 0,1,2)_ magnetic nanoparticles (MNPs) (APTMS: (3-aminopropyl)trimethoxysilane), presenting a core shell structure and previously prepared by the authors, was used in this system. After optimization of the reaction conditions, the scope of the presented procedure was examined for the reaction between various aryl(naphthyl) halides and phenylboronic acid. The current strategy afforded relatively short and acceptable reaction times and good-to-excellent yields of the pure biaryl products (Table 11). The reaction rate was found to be strongly influenced by the presence of electron-donating and electron-withdrawing groups, the latter presenting a higher rate rather comparatively. Furthermore, the TON and TOF values were obtained up to 481 and 2884, respectively, for the mentioned Suzuki–Miyaura cross-coupling reaction.

### 3.4. Ionic Liquids

Ionic liquids (ILs) are understood as liquids composed of poorly coordinated ions with a melting point below 100 °C that have recently attracted great interest as a “greener” alternative to conventional organic solvents due to their notable physicochemical properties [51]. These include thermal stability; nonflammability; recyclability; low vapor pressure; and catalytic properties, such as nonflammability, nonvolatility, polarity, and stability [70]. The remarkable properties of ILs can be substantially modified by changing the cationic and/or the anionic components, which can give rise to ILs with specific properties via numerous combinations of cations and anions [71]. One of the most extensively studied class of ILs is based on imidazolium cations with an appropriate counter anion, which are known to support many organic transformations. In view of these attributes, ILs are increasingly being used as popular reaction media in synthesis, analysis, catalysis, and separation, as well as medicine and pharmaceuticals, as efficient and sustainable media and, not surprisingly, have been used in cross-coupling reactions as efficient and sustainable media [10]. 

In 2015, Patil and coworkers described the use of dual-functionalized task-specific hydroxyl functionalized IL as an excellent promoter in the Pd-catalyzed Suzuki–Miyaura cross-coupling of aryl halides with arylboronic acids in water [72]. The IL cation with hydroxyl functionality induced a reaction in water, acting as a reducing agent, whereas the anion with prolinate functionality served as the ligand and stabilized and/or activated the in-situ generation of PdNPs. The generated PdNPs exhibited excellent catalytic activity towards the Suzuki cross-coupling of aryl halide with aryl boronic acid in water, without the need of a phosphine ligand (Table 12). 

Strikingly, the coupling of less activated aryl chlorides also proceeded smoothly with aryl boronic acid. The aqueous system containing ionic liquid along with PdNPs presented a good recyclability, up to seven times, without a noticeable loss of catalytic activity, showing potential for industrial applications.

Hejazifar and coworkers reported the design and properties of surface-active ILs able to form stable microemulsions with heptane and water and their application as a reaction media for Pd-catalyzed cross-coupling reactions [73]. The application of the microemulsions as a reaction media resulted in high reactivity even at a low catalyst loading, with excellent yields > 90% for most products resulting from the coupling of aryl halides or boronic acids with electron-deficient substituents. The gathered data demonstrated the dual role of IL of a surfactant and ligand not limited to the formation of a suitable reaction media, providing the outstanding reactivity reported. The catalytic system behavior allowed the simple and successful product separation and catalyst recycling. 

**Figure 11:**
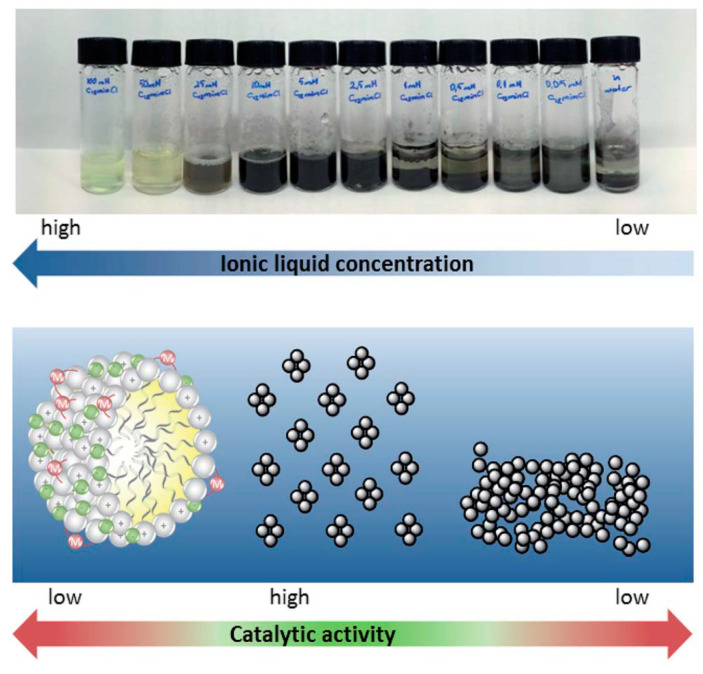
Concentration dependence of Pd-metallomicelles and PdNPs in an aqueous-ionic liquid (IL) micellar solution. Conditions: 2 mL (C1_2_mimCl) 1 solution, 0.02 mmol Pd_2_(allyl)_2_Cl_2_, and 0.02 mmol K_2_CO_3_ 60 min at 80 °C under air. Reproduced with the permission of reference [74], Copyright 2017 The Royal Society of Chemistry.

The effects of the IL concentration on the catalytically active species towards Pd-catalyzed cross-coupling reactions was studied by Taskin et al. [74]. A series of surface-active IL were applied as additives in the Heck reaction of ethyl acrylate and iodobenzene, obtaining high yields (> 90%) in water, pointing out the IL concentration as the key factor affecting the formation of the catalytically active species in the reaction, the morphology, and chemical state of the Pd species (Figure 11). 

At higher concentrations, the formation of a *N*-heterocyclic carbene complex was observed, while, at lower surfactant concentrations, the rapid decomposition of metallomicelles into catalytically active Pd(0) nanoclusters occurred, a unique behavior only observed for amphiphilic IL that differs significantly from dimethylimidazolium-based IL or from conventional surfactants. The dual role of imidazolium-based surfactants as templates for NP formation through NHC carbene complex formation, as well as stabilizers and surfactants for a consecutive reaction, was postulated by the authors.

The Hayouni research group used their previous expertise in the use of bio-sourced ILs as solvents in hydrogenation processes to explore the potentialities of these ILs in the Heck reaction [75]. Bio-sourced ammonium and phosphonium carboxylate ILs obtained from l-lactic acid or l-malic acid were easily synthesized and used as the solvent for the Heck coupling reaction between iodoarene or halogenoaromatics with *tert*-butylacrylate used PdCl_2_ as catalyst. 

With the use of NaHCO_3_, good conversion and selectivity were obtained using tetrabutylammonium (TBA) ILs, presenting better results than those for a typical coupling media as the usual organic solvents or commercial ILs, although the catalytic system could not be recycled. The replacement of the base by triethylamine NEt_3_, a relative strong homogeneous base, afforded good efficiency, with the advantageous possibility of recycling of the catalytic system without a loss of reactivity up to five times (Table 13).

**Figure 12:**
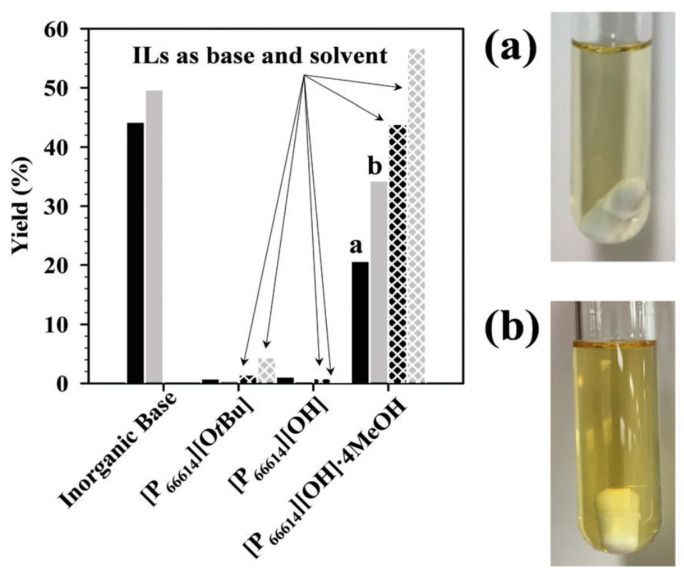
(**Left**)**:** Effect of (P_66614_)^+^ bases vs. inorganic bases on Pd-catalyzed Buchwald–Hartwig amination (BHA) (black) and Suzuki–Miyaura coupling (SMC) (grey). Solid bars: 1,4-dioxane as the solvent. Crosshatched bars: ILs as both the base and solvent. Inorganic base: KO*t*Bu for BHA and K_2_CO_3_ for SMC. (**Right**): Final reaction mixtures (with magnetic stir bar) for BHA (**a**) and SMC (**b**) using (P_66614_)(OH)_4_MeOH as the base and dioxane as the solvent. Reproduced with the permission of reference [76], Copyright 2018 The Royal Society of Chemistry.

In 2018, solid salt precipitation issues in cross-coupling reactions were addressed by combining basic anions with the trihexyl(tetradecyl)phosphonium ((P_66614_)^+^) cation, ensuring an IL by-product [76]. Among all synthesized basic salts, the novel IL base (P_66614_)(OH)_4_MeOH showed the highest basic strength, being the most efficient in the Pd-catalyzed Suzuki–Miyaura cross-coupling reaction (Figure 12). No precipitate was observed after the catalytic run due to the generation of the room temperature IL (P_66614_)Cl. In addition, an easy recycling of the generated IL was predicted simply by elution with MeOH on an ion exchange column, since (P_66614_)Cl was used in the synthesis of the active base ((P_66614_)(OH)_4_MeOH) in an ion-exchange process.

The enhanced catalytic reaction rates for the Suzuki–Miyaura cross-coupling reaction was highlighted by the authors as another advantage of the basic salt being an IL itself, when (P_66614_)(OH)_4_MeOH employed as both the base and solvent was preferred over the use of 1,4-dioxane as the solvent.

**Scheme 10:**
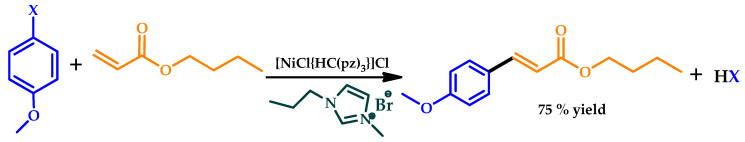
Heck reaction of 4-bromoanisole and butyl acrylate in 1-butyl-3-methylimidazolium bromide, catalyzed by [NiCl{κ^3^-HC(pz)_3_}]Cl (pz = pyrazol-1-yl) [77].

In 2019, Matias et al. used, for the first time, a C-scorpionate Ni(II) complex (NiCl(κ^3^-HC[pz]_3_))Cl (pz = pyrazol-1-yl) as a catalyst for the MW-assisted Heck–Mizoroki C–C cross-coupling in IL medium, obtaining good yields of the corresponding products [77]. The replacement of common organic solvents by suitable room temperature ILs as reaction medium addressed both the recyclability of the catalyst and sustainability using greener solvents (Scheme 10). Furthermore, the use of the IL allowed an easy separation of the product from the catalyst, since, at high temperatures, the IL dissolves all components of the reaction mixture, but, at room temperature, the substrates and products form a second phase. The stability of the catalyst in the IL allowed its recycling and reuse for several consecutive catalytic cycles with preserved activity.

### 3.5. Deep Eutectic Solvents

The concept of deep eutectic solvents (DES) was introduced by Abbott and coworkers in 2003 [78] with the deployment of ChCl:ZnCl_2_ acidic solvent in several reactions as a green alternative for ILs. The concept of DES is, however, quite different from that of traditional ILs, since they are not entirely composed of ionic species. DES are nowadays usually understood as combinations of two or three safe and inexpensive components that are able to establish hydrogen bond interactions with each other to form an eutectic mixture that presents a lower melting point than either of the individual components [12,79]. DES constitute important alternatives to conventional organic solvents due to their interesting properties and benefits, such as an attractive low price, no flammability, ease synthetic accessibility, and benign and safe nature, along with recyclability and biodegradability and a low ecological footprint. The most widely deployed DES are prepared by mixing a hydrogen bond acceptor, exemplified by an inexpensive quaternary ammonium salt such as choline chloride (ChCl, included in the so-called vitamin B4), with a hydrogen bond donor such as sugars, organic and amino acids, urea (most of them are from renewable resources), or glycerol (a biowaste). The use of DES allows to overcome drawbacks of ILs, such as a complicated synthesis procedure, difficult high purity, and sensitivity to air and moisture. For example, the melting points of the DES are lower than the melting points of the ILs. In addition, DES consists of relatively cheaper compounds when compared to IL components. DES allows the solvation of various solutes in high concentrations much higher than their solubility in water. When compared to the conventional solvents, DES are recognized as highly soluble, relatively less toxic, thermally stable, biodegradable, nonflammable, and nonvolatile [12,80].

DES are now a matter of growing interest both at the academic and industrial levels. Extensive and valuable reports have been published highlighting the beneficial impacts of DES in organic reactions, playing a dual role of DES as a solvent and catalyst for many chemical processes [10,12]. Although, the application of neoteric media in metal-catalyzed reactions has witnessed a remarkable growth in recent years, fewer reports of catalytic systems regarding cross-coupling reactions carry out in DES media are available in the literature. 

**Figure 13:**
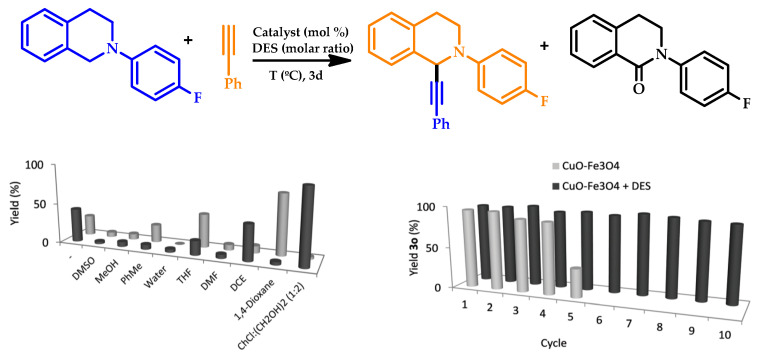
(**Top**) Reaction scheme of 2-(4-fluorophenyl)-1,2,3,4-tetrahydroisoquinoline and phenylacetylene. (**Bottom left**) Obtained yield in different solvents for the products of the reaction shown on top. (**Bottom right**) Recycling of the CuO–Fe_3_O_4_ catalyst and CuO–Fe_3_O_4_ and deep eutectic solvent (DES). Reproduced with the permission of reference [81], Copyright 2016 The Royal Society of Chemistry.

In 2016, the synthesis of different tetrahydroisoquinolines using a DES (choline chloride:ethylene glycol) and a catalyst based on magnetite-supported copper(II) oxide was explored by Marset and coworkers [81]. The presence of a DES medium proved to be essential for the reaction to occur after the testing of other VOC solvents as the reaction medium, being also important for minimizing the lactam formation (Figure 13, left). Excellent results were obtained for the aryl group substituent, bearing both electron-withdrawing or electron-donating groups), whereas, for phenyl derivatives, moderate yields were obtained. A direct proportional relationship was also found between the conductivity of the DES medium and the yield obtained. Moreover, the amount of copper in the catalyst was the lowest loading ever reported [82,83,84,85].

The mixture (solvent and catalyst) was reused for ten consecutive cycles without any significant loss of activity with this highly sustainable protocol, with water as the only stoichiometric waste, demonstrating the high recyclability of the system (Figure 13, right). Upon catalyst recovery by magnetic decantation, the obtained yield demonstrated a sharp decrease after four reaction cycles.

In 2017, the same research group reported the synthesis of cationic pyridiniophosphine ligands to develop DES-compatible catalytic systems. The ligands were successfully employed to different Pd-catalyzed cross-coupling reactions—namely, Suzuki–Miyaura, Sonogashira, or Heck couplings. Optimization studies were performed to determine the best catalyst for the different reaction. Furthermore, the scope of the cross-coupling reactions was explored, obtaining moderate-to-good yields in general. The cationic phosphines enhanced the catalytic activity of Pd in this polar medium. Moreover, the recyclability of these processes was also investigated, allowing the reuse of both catalyst and DES up to five times without a significant loss of catalytic activity. The DES choline chloride:glycerol (1:2) was used as sustainable medium for the Pd-catalyzed Suzuki–Miyaura cross-couplings between (hetero)aryl halides (Cl, Br, and I) and mono- and bifunctional potassium aryltrifluoroborates. The reaction proceeded efficiently and chemoselectively in the air and under mild conditions, with a low catalyst loading (1 mol%) and employing Na_2_CO_3_ as the base, without further additional ligands [86]. Overall, valuable biaryls and terphenyl derivatives were obtained in excellent yields (>98%). The DES, catalyst, and base were easily recovered and reused up to six cycles, with an E_factor_ as low as 8.74. The proposed methodology was also applied by the authors to the synthesis of two nonsteroidal anti-inflammatory drugs—namely, Felbinac and Diflunisal.

**Figure 14:**
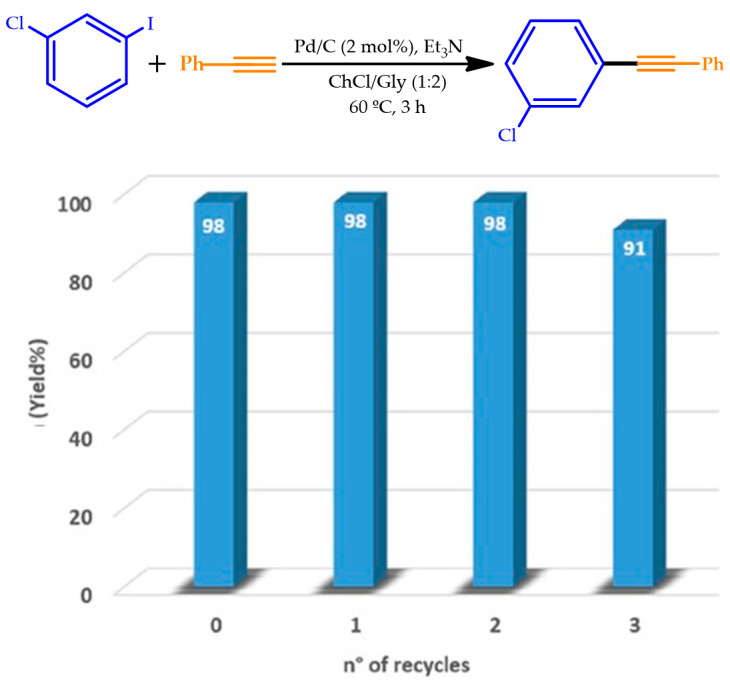
Recycling of Pd/C (2 mol%) and DES in the coupling reaction between 1-chloro-3-iodobenzene and phenylacetylene. Adapted with the permission of reference [87], Copyright 2020 Wiley VCH.

The above-mentioned DES, ChCl:Gly (1:2), was found to promote Sonogashira cross-couplings using the commercially available Pd/C in the absence of external ligands. Heteroaryl iodides were effectively coupled with both aromatic and aliphatic alkynes under heterogeneous conditions, affording yields in the range 50–99% for 3 h at 60 °C by Messa et al. [87]. This catalytic system was also active towards the Pd-catalyzed coupling electron-poor and electron-rich (hetero)aryl iodides, known to be poorly reactive, with several aromatic alkynes, presenting a broad substrate scope and affording yields ranging from 54% to 99%. The catalyst and the DES used were successfully recovered and reused up to four times (Figure 14), presenting an E_factor_ as low as 24.4. A small change in the ligand structures, from phosphines to cationic pyridiniophosphines, was enough to keep the excellent results in this DES medium, such as those obtained in classical organic solvents. Moreover, the catalyst load increased up to 5 mol% with the use of CuI (20 mol%) as the cocatalyst, demonstrating the feasibility of the difficult SH Pd-catalyzed cross-couplings of alkyl-, trimethylsilyl-, and hydroxybenzyl-substituted alkynes, obtaining yields in the range of 63–93%.

In 2019, Saavedra et al. developed a novel, versatile, and DES-compatible bipyridine Pd complex as a general precatalyst for several cross-coupling reactions [88]. The application of the prepared Pd precatalyst to the Hiyama, Suzuki–Miyaura, Heck–Mizoroki, and Sonogashira cross-coupling reactions in DES under aerobic conditions demonstrated a high catalytic activity. The same Pd precatalyst was able to catalyze a broad number of cross-coupling reactions, keeping the excellent results obtained in classical organic solvents given the hydrogen bond capacity of the ligand. 

The catalyst and DES system were easily and successfully recycled up to three or five consecutive cycles, depending on the cross-coupling reaction, without a significant loss of activity. Overall, the authors highlighted the great applicability, versatility, and sustainability of this system.

**Figure 15:**
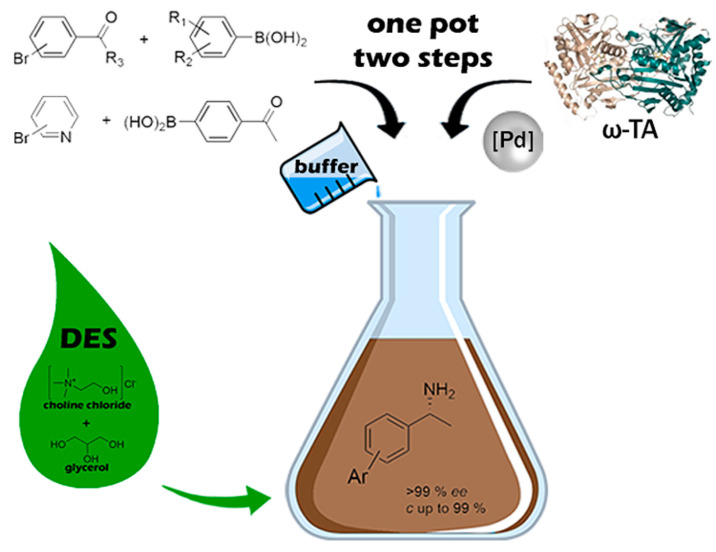
Strategy proposed for the one-pot synthesis of biaryl-substituted amines by combining palladium and enzyme catalysis in DES. Reproduced with the permission of reference [89], Copyright 2019 American Chemical Society.

The first application of DES in the bioamination of ketones was devised by Paris and colleagues [89]. The stability of the amine transaminases (ATAs) was confirmed in DES-buffer mixtures up to 75% (*w/w*) neoteric solvent, affording a good catalytic performance with high conversions (Figure 15). The solubility of DES allowed the metal-catalyzed step at 200-mM loading of the substrate and the following biotransformation at 25 mM. A chemoenzymatic cascade toward enantiopure biaryl amines was therefore efficiently established owing to the unique properties of DESs. The authors presented an excellent proof of concept for the unparalleled enzymatic activity of ATAs, highlighting the practical value of biorenewable solvents in organic synthesis. 

## 4. Conclusions

The deployment of unconventional conditions for cross-coupling reactions, whether alternative energy inputs, as well as new green unconventional media individually or combined, has brought tremendous benefits and innovative approaches to synthetic chemistry. The use of these groundbreaking approaches is in complete agreement with the principles of green chemistry through their numerous advantages: change of reactivity, improvement of yields and selectivity, reduction of reaction time, energy saving, and waste minimization. 

Regarding alternative activation energy for cross-coupling reactions, important aspects must be taken into consideration. The suggested approaches should be simple, without being too specific, with an underlying mechanism and a systematic characterization of operational parameters and a description of the experimental conditions of the MW, US, and mechanochemistry-assisted reactions to facilitate comparisons between studies. The coupling of different technologies with other unconventional media or methods of activation and the possible synergetic effects arising thereof, as well as new fields of applications, should also be highlighted. The possibility of scaling-up the lab-scale methodologies that provide excellent results would be the true realization of the sustainability goals aimed for by the authors. Considering the novelty witnessed in the scrutinized publications, a bright future for the development of sustainable cross-coupling reactions is envisioned in the years to come. 

## Figures and Tables

**Table 1:** Suzuki cross-coupling reaction of phenylboronic acid with various aryl halides under optimized reaction conditions under microwave (MW) irradiations [29].

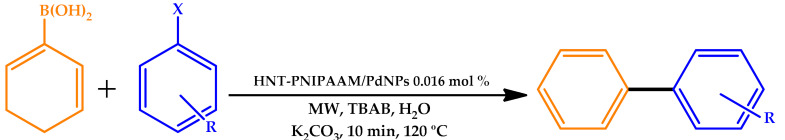			
Ar-X	Conversion (%)	TON	TOF (h^−1^)
4-bromoacetophenone	>95	6250	37,500
4-iodoanisole	85	5310	31,870
4-bromobenzaldheyde	94	5880	35,250
4-bromoanisole	85	5310	31,880
3-bromobenzaldheyde	82	5130	30,750
4-iodoacetophenone	94	5880	35,250
2-iodotoluene	73	4560	27,380

Turnover number (TON) calculated as moles of substrate converted/moles of Pd. Turnover frequency (TOF) calculated as TON/hours. HNT: halloysite nanotube; PNIPAAM: HNT-poly(N-isopropylacrylamide); PDNPs: Pd nanoparticles, TBAB: tetrabutylammonium bromide.

**Table 2:** Study of the effect of ultrasonic radiation on the Sonogashira reaction [41].

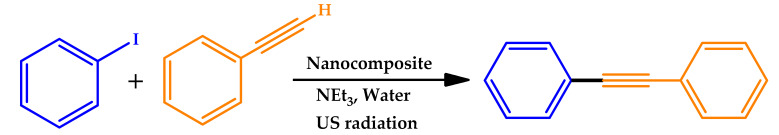		
Power (W)	Time (min)	Yield (%)
Silent	120	-
35	60	81
40	50	87
45	35	93
50	25	98
55	25	98

Reaction conditions: aryl halide (1 mmol), phenyl acetylene (1.2 mmol) GO@SiO_2_-HMTA-Cu(I) and NEt_3_ (3 mmol) in water. GO: graphene oxide; HMTA: hexamethylenetetramine.

**Table 3:** Study of the effects of ultrasonic irradiation on the Mizoroki-Heck cross-coupling [42].

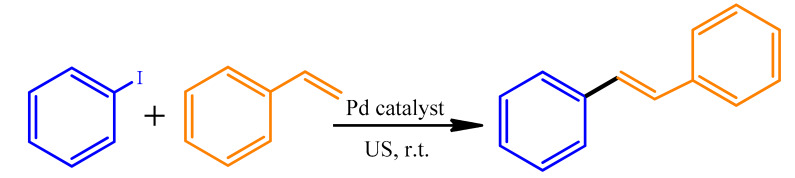		
Power (W)	Time (min)	Yield (%)
25	30	61
30	26	73
35	21	85
40	15	91
45	8	99
50	8	99

Reaction conditions: Iodobenzene (1 mmol), styrene (1.2 mmol) catalyst (20 mg), and NEt_3_ (2 mmol) in DMF.

**Table 4:** Investigation on the effects of varying the mechanochemical parameters [47].

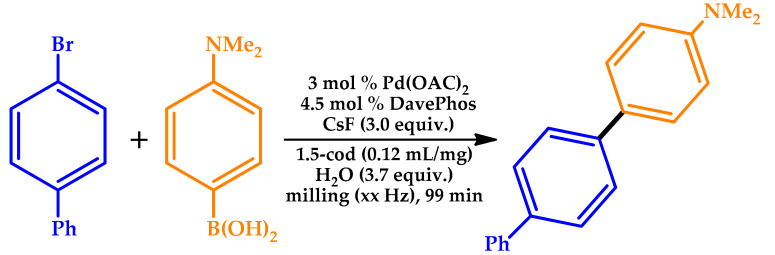			
Frequency (Hz)	Number of Balls	Ball Size` (mm)	Yield (%)
10	1	5	70
15	1	5	75
20	1	5	70
25	1	5	97
30	1	5	99
25	1	3	90
25	2	5	97

Reaction conditions: Pd(OAc)_2_ (0.009 mmol), DavePhos (0.0135 mol), CsF (0.9 mmol), H_2_O (20 μL), and 1,5-cod (0.12 μL/mg) in a stainless-steel ball milling jar (1.5 mL).

**Table 5:** Reuse of the PdNPs/MWCNT catalyst in the solid-phase Suzuki–Miyaura process [49].

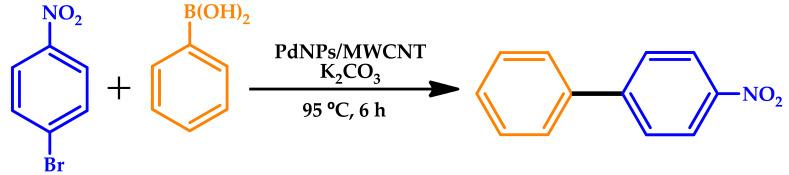							
Conversion%	Cycle						
	1	2	3	4	5	6	7
0.1 mol% of the catalyst	95	75	94	84	100	98	100
0.5 mol% of the catalyst	79	80	88	86	96	97	93

Reaction conditions: 1 mmol of aryl halide, 1.2 mmol of phenylboronic acid, 1.2 mmol of K2CO3, and 95 °C for 6 h. MWCNT: multi-walled carbon nanotubes.

**Table 6:** Mechanochemical Suzuki—Miyaura cross-coupling reactions of bromobenzene with phenylboronic acid [50].

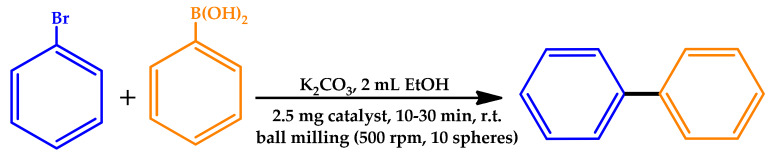		
Catalyst	Time	Yield (%)
AC-Pd1	10	27
	30	30
AC-Pd2	10	41
	30	49
	180	69
CNT-Pd1	10	37
	30	29
CNT-Pd2	10	32
	30	50
Pd(OAc)_2_	30	75

**Table 7:** Catalytic activity of the guar gum (GG)-Pd catalyst in Suzuki–Miyaura coupling reactions [53].

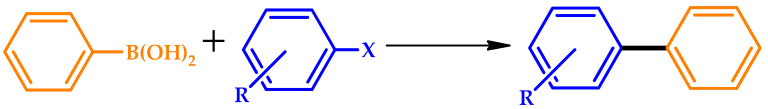			
R	X		
	I	Br	Cl
4-OCH_3_	98	88	56
3-NO_2_	87	85	70
3-CH_3_	62	59	33
4-CH_3_	71	69	42

**Table 8:** Suzuki cross-coupling reactions between phenylboronic acid and various aryl halides catalyzed by PdNP-polumeric ionic liquid (PIL) [58].

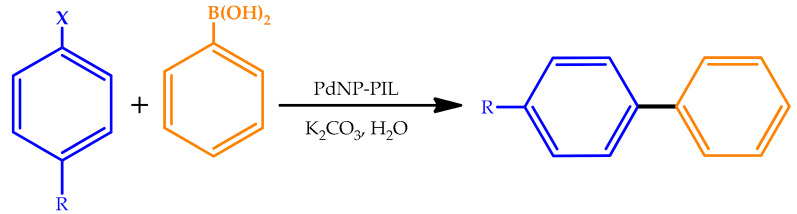		
R	X	Yield (%)
H	I	98
Me	I	93
MeO	I	99
NO_2_	I	99
CN	I	97
Me	Br	70
MeO	Br	99
NO_2_	Br	92
CN	Br	98
Me	Cl	23
MeO	Cl	17
CN	Cl	16

**Table 9:** Reduction of Pd loading for the ligand-free Suzuki–Miyaura reaction between 4-methoxyphenylboronic acids and bromobenzene [60].

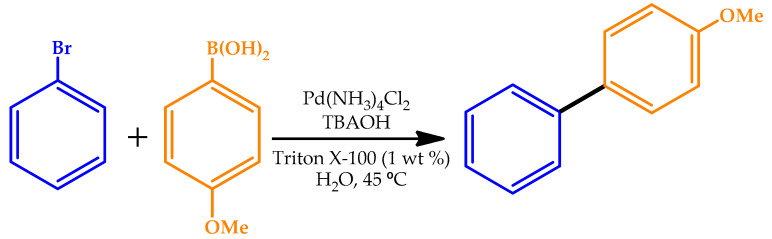					
Pd Loading (ppm)	TBAOH (equiv.)	t (h)	Yield (%)	TON	TOF (h^−1^)
30	2	2	>99	33,000	16,500
30	1	2	90	30,000	15,000
30	4	6	75	25,000	4100

Reaction conditions: bromobenzene (1 mmol), 4-methoxyphenylboronic acid (1.2 mmol), TBAOH (1–4 mmol), Triton X-100 (0.1 mL), and H_2_O (7 mL), 45 °C, air.

**Table 10:** Catalytic results for the Heck reaction [68].

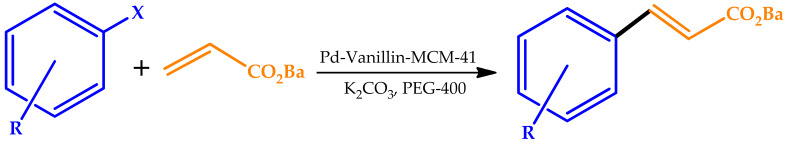			
Solvent	Base (eq.)	Catalyst (mol%)	Yield (%)
PEG-400	K_2_CO_3_	0.53	72
PEG-400	K_2_CO_3_	0.71	91
PEG-400	K_2_CO_3_	0.88	91
EtOH	K_2_CO_3_	0.71	-
H_2_O	K_2_CO_3_	0.71	75
DMF	K_2_CO_3_	0.71	83
PEG-400	Et_3_N	0.71	90
PEG-400	NaHCO_3_	0.71	75
PEG-400	NaHCO_3_	0.71	88
PEG-400	NaHCO_3_	0.71	35

Reaction conditions: aryl halide (1 mmol), *n*-butyl acrylate (1.2 mmol), and base (3 mmol), 120 °C, 25 min. PEG-400: poly(ethylene glycol).

**Table 11:** Suzuki–Miyaura cross-coupling reaction in the presence of Fe_3_O_4_@APTMS@Cp_2_ZrCl_x(x = 0, 1, 2)_ as a Pd-free nanocatalyst in PEG-400 [69]. APTMS: (3-aminopropyl)trimethoxysilane.

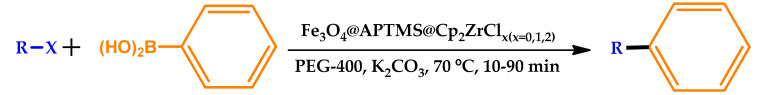			
R	Yield (%)	TON	TOF (h^−1^)
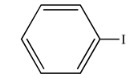	98	481	1424
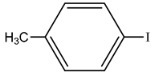	96	471	628
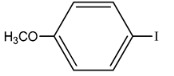	83	407	444
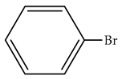	95	466	1398
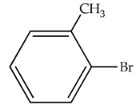	80	392	336
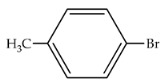	83	407	543
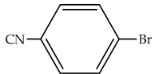	97	476	1903

**Table 12:** Screening of ionic liquids for the Suzuki–Miyaura coupling [72].

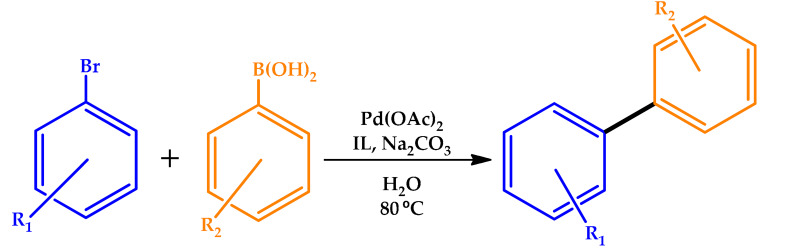			
IL	Loading (mol%)	Time (h)	Isolated Yield (%)
[BMIM][Cl]	20	2	65
[BMIM][BF4]	20	1	76
[BMIM][Pro]	20	1	80
[HEMPy][Cl]	20	2	80
[HEMPy][BF4]	20	1	84
[HEMPy][Pro]	20	1	98
1-proline	20	2	50
--------	20	2	40
[HEMPy][Pro]	10	1	84
[HEMPy][Pro]	15	1	90
[HEMPy][Pro]	25	1	98
[HEMPy][Pro]	30	1	98

(BMIM) = 1-butyl-3-methylimidazolium, (HEMPy) = 1-(2-hydroxyethyl)-1-methylpyrrolidinium, (Cl) = chloride, (BF4) = tetrafluoroborate, (PF6) = hexafluorophosphate, and (Pro) = prolinate.

**Table 13:** Catalytic results for the Heck reaction with organic solvents and ionic liquids (ILs) with NEt_3_ as the base [75].

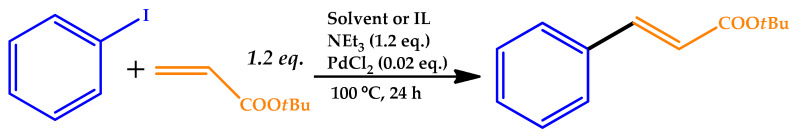			
Solvent or ILs	Cycle	Conversion (%)	Selectivity (%)
toluene	1	1	100
water	1	30	100
DMSO	1	76	80
TBP Br	1	47	100
TBA Cl	1	99	100
	2	100	100
	3	61	100

Reactions conditions: 1.2 mmol of iodobenzene and 600 mg of solvent. TBP: tetrabutylphosphonium, TBA: tetrabutylammonium.
